# Long-term outcomes of conservative treatment and endovascular treatment in patients with symptomatic spontaneous isolated superior mesenteric artery dissection: a single-center experience

**DOI:** 10.1186/s12872-020-01532-y

**Published:** 2020-05-29

**Authors:** Leyin Xu, Jiang Shao, Daming Zhang, Chenyang Qiu, Jingjing Wang, Kang Li, Lijing Fang, Xin Zhang, Jinsong Lei, Zhichao Lai, Jiangyu Ma, Yanying Yu, Xiaoxi Yu, Fenghe Du, Wanting Qi, Junye Chen, Bao Liu

**Affiliations:** 1 Department of Vascular Surgery, Peking Union Medical College Hospital, No. 1 Shuaifuyuan, Dongcheng District, Beijing, 100730 China; 2 Department of Radiology, Peking Union Medical College Hospital, No. 1 Shuaifuyuan, Dongcheng District, Beijing, 100730 China

**Keywords:** Isolated superior mesenteric artery dissection, Conservative treatment, Endovascular treatment, Treatment outcome, Long-term follow-up

## Abstract

**Background:**

Spontaneous isolated superior mesenteric artery dissection (SISMAD) is a rare vascular disorder, and the treatment strategies remain controversial. This study aimed to compare outcomes of conservative and endovascular treatments in symptomatic patients with SISMAD.

**Methods:**

Forty-two consecutive SISMAD patients who were admitted to a single center between October 2009 and May 2018 were enrolled in this study. Based on their symptoms, 15 had conservative treatment, and 27 had endovascular treatment. The baseline characteristics, treatments, and follow-up results of the conservative group and endovascular group were analysed.

**Results:**

The rates of symptom relief were 93.3% in the conservative group and 96.3% in the endovascular group. The procedure-related complications in the endovascular group included one case of pseudoaneurysm formation in the left brachial artery. During the follow-up period (median 28.5 months), a higher proportion of patients in the conservative group had symptom recurrence (42.9% in the conservative group versus 4.8% in the endovascular group, *p* < 0.001). Four patients in the conservative group and one patient in the endovascular group had additional endovascular intervention during follow-up. Compared with the conservative group, patients in the endovascular group had statistically significantly longer symptom-free survival (*p* = 0.014) and a higher rate of superior mesenteric artery (SMA) remodeling (*p* < 0.001).

**Conclusions:**

For symptomatic SISMAD, endovascularly treated patients had a lower rate of symptom recurrence and a higher rate of SMA remodeling in the long term. Prospective, multi-center studies are needed to confirm the long-term outcomes of both treatments.

## Background

Spontaneous isolated superior mesenteric artery dissection (SISMAD) is a rare vascular disorder. First reported in a case series by Bauersfeld in 1947 [1], it is increasingly recognized using imaging techniques. Conservative treatment, endovascular treatment, and open surgical repair are the main treatment strategies. Open surgery is indicated in patients who have intestinal necrosis [2]. As recommended by the European Society of Vascular Surgery (ESVS) guidelines, conservative treatment is the first-line therapy for symptomatic patients [3]. However, conservative treatment as an initial treatment had unsatisfactory results in some symptomatic patients. Garrett reported that the failure rate of initial conservative treatment was approximately 16% in the symptomatic SISMAD patients, and some severe complications occurred [4]. During the follow-up period, 20% of the patients with initial conservative treatment ultimately underwent endovascular treatment [3]. Follow-up imaging showed that only 25% of the patients with conservative treatment achieved complete remodeling, and 12% had morphological progression [5]. Conservatively treated patients might be at risk of symptom recurrence and disease progression in the long term.

The effectiveness and safety of endovascular treatment have been proven in previous studies [6–8]. During mid-term follow-up, endovascular treatment was associated with a high primary stent patency rate and a low cumulative event-free survival rate [2]. According to the ESVS guidelines, endovascular treatment could be indicated when patients are not responding to conservative treatment or with suspicion of bowel ischemia [3]. However, due to its minimal invasiveness and favorable outcomes in the long term, endovascular treatment might be the first-line treatment as well, and further studies are needed.

The long-term outcomes of conservative treatment and endovascular treatment in symptomatic SISMAD patients remain to be compared. In this study, we summarize our experience with both treatments, with the goal of determining the optimal treatment strategy.

## Methods

### Study population

This study retrospectively analysed the data from 42 consecutive SISMAD patients who were admitted to the study hospital between October 2009 and May 2018. The diagnoses of SISMAD were based on computed tomography angiography (CTA) or magnetic resonance angiography. Patients were excluded if they 1) were asymptomatic; 2) had aortic dissection, systemic vasculitis, or other autoimmune diseases; or 3) were treated with open surgery.

Patients were divided into a conservative group (15 patients) and an endovascular group (27 patients) according to their first treatment in the study hospital. Information including baseline characteristics, treatments, and outcomes was collected from the medical records. Yun’s classifications were used for describing the features of dissections (Type I: dissections with patent true lumen and false lumen and revealing both entry and re-entry sites; Type IIa: dissections with patent true lumen but no re-entry site in the false lumen; Type IIb: dissections with patent true lumen and thrombosed false lumen; and Type III: dissections with completely occluded superior mesenteric artery (SMA)) [11]. All radiographic findings were based on CTA or digital subtraction angiography.

### Treatment strategies

The treatment algorithm was based on the clinical features of each patient. If a patient had a history of symptom recurrence after conservative treatment or a ruptured dissecting aneurysm or if he or she was suspected of having intestinal ischemia, endovascular treatment was indicated. Other patients had medical treatment only. If a patient had persistent symptoms or signs after at least 7 days of conservative treatment, endovascular intervention was considered.

Conservative treatment included strict blood pressure control, bowel rest, antiplatelet therapy, and anticoagulation therapy. Fasting was used for patients with severe symptoms or signs, and diets were resumed as soon as the symptoms and signs disappeared. The antiplatelet therapy regimen included aspirin, clopidogrel, or both, and the course was 3 to 6 months. Anticoagulation medications, including warfarin, low-molecular-weight heparin, or rivaroxaban, were used when thrombus existed.

Stent placement (usage of bare metal stents) was the main method of endovascular intervention. The use of a single stent or overlapping stents depended on the lengths of the lesions. Balloon angioplasty was used for the lesions with severely stenosed or completely occluded true lumens. Patients with successful intervention took either aspirin alone, or aspirin and clopidogrel for at least 6 months. Patients with thrombus took aspirin and one anticoagulant after stent placement.

### Follow-up

Patients were required to attend outpatient clinic visits at 3 months, 6 months, 1 year, and annually thereafter. Both clinical and radiographic evaluations were applied. CTA and Doppler ultrasonography were the main methods used to assess the lesions. Complete remodeling was defined as the complete disappearance of the dissection in the SMA, without arterial stenosis or thrombus. For patients with recurrent symptoms, laboratory and radiographic examinations could help identify the causes. When other causes were excluded, revascularization was considered in these patients if they had severe stenosis or occlusion in the SMA.

### Statistics

Student’s *t* test, the Wilcoxon rank-sum test, and Fisher’s exact test were used to compare the results of the study population. Kaplan-Meier analysis was performed to estimate the event-free survival rate, and a log-rank test was applied to compare the two groups. The appearance of any symptoms related to SISMAD (relapsing abdominal pain and other abdominal discomfort) was defined as an event. A *p* value < 0.05 was considered to be statistically significant in all analyses. Statistical analyses were performed using IBM SPSS Statistics software, version 22.

## Results

### Clinical characteristics

Baseline characteristics, including age, sex, comorbidities, clinical manifestations, and laboratory and radiographic results, are summarized in Table 1. The mean age of the patients was 52.3 ± 7.3 years, and only two patients (1 in the conservative group and 1 in the endovascular group) were women. Comorbidities included hypertension, diabetes mellitus, and hyperlipidemia, and smoking had a high prevalence (16/42, 38.1%) among the patients. Abdominal pain was the chief complaint of 38 (90.5%) patients. Intestinal obstruction occurred in 4 patients, and all these patients underwent endovascular treatment. Most patients showed normal results in white blood cell count, high-sensitivity C-reactive protein, and erythrocyte sedimentation rate. However, hyperhomocysteinemia was common in these symptomatic SISMAD patients. There were no statistically significant differences in any of these characteristics between the two groups.

**Table 1:** Baseline characteristics of patients with SISMAD

	Total (*N* = 42)	Conservative group (*n* = 15)	Endovascular group (*n* = 27)	*p* value
Age, mean ± SD, years	52.3 ± 7.3	51.4 ± 6.6	52.8 ± 7.7	0.563
Men, n (%)	40 (95.2)	14 (93.3)	26 (96.3)	1.000
Comorbidities, n (%)				
Hypertension	23 (54.8)	10 (66.7)	13 (48.1)	0.337
Diabetes mellitus	6 (14.3)	2 (13.3)	4 (14.8)	1.000
Hyperlipidemia	19 (45.2)	7 (46.7)	12 (44.4)	1.000
Smoking	16 (38.1)	5 (33.3)	11 (40.7)	0.746
Clinical manifestations, n (%)				
Abdominal pain	38 (90.5)	14 (93.3)	24 (88.9)	1.000
Nausea and vomiting	11 (26.2)	5 (33.3)	6 (22.2)	0.481
Diarrhea	2 (4.8)	1 (6.7)	1 (3.7)	1.000
Abdominal distention	7 (16.7)	1 (6.7)	6 (22.2)	0.390
Hematochezia	3 (7.1)	1 (6.7)	2 (7.4)	1.000
Intestinal obstruction	4 (9.5)	0	4 (14.8)	0.279
Laboratory findings				
WBC, mean ± SD, 10^9^/L	6.4 ± 2.3	6.2 ± 1.6	6.6 ± 2.7	0.579
hsCRP, median(IQR), mg/L	1.17 (0.48, 6.72)	0.69 (0.48, 8.47)	1.17 (0.47, 6.41)	0.983
ESR, median(IQR), mm/h	11.0 (2.5, 21.0)	8.0 (2.0, 12.0)	12.5 (5.8, 22.25)	0.525
HCY > 15 μmol/L, n (%)	12 (60.0)	6 (75.0)	6 (50.0)	0.373
Radiographic findings				
True lumen stenosis (> 70%), n (%)	17 (40.5)	6 (40.0)	11 (40.7)	1.000
Occlusion in the true lumen, n (%)	9 (21.4)	3(20.0)	6(22.2)	1.000
The distance from the SMA ostium to the beginning of the dissection, mean ± SD, mm	19.3 ± 12.2	14.0 ± 6.3	21.2 ± 13.2	0.184
Length of the dissection, mean ± SD, mm	43.0 ± 21.1	42.6 ± 19.3	43.2 ± 22.2	0.948
Dissection aneurysm, n (%)	26 (60.5)	10 (66.7)	15 (55.6)	0.531
Branch involvement, n (%)	24 (57.1)	5 (33.3)	19 (70.4)	0.027
Yun’s classification I, n (%)	1 (2.4)	0 (0)	1 (3.7)	
Yun’s classification IIa, n (%)	15 (35.7)	5 (33.3)	10 (37.0)	
Yun’s classification IIb, n (%)	19 (45.2)	7 (46.7)	12 (44.4)	
Yun’s classification III, n (%)	7 (16.7)	3 (20.0)	4 (14.8)	

*WBC* white blood cell, *hsCRP* high-sensitivity C-reactive protein, *ESR* erythrocyte sedimentation rate, *HCY* homocysteine, *IQR* interquartile range

The radiographic findings of both groups were compared. Severe stenosis and complete occlusion in the true lumens were common in the patients. Most of the dissections were located close to the SMA ostium, and the mean distance was 19.3 ± 12.2 mm. The mean lengths of the dissections were similar between the two groups. Dissection aneurysms (at least 1.5 times larger than the normal SMA) and branch involvement were frequently observed. Because of the high prevalence of thrombus, Yun’s type IIb (19, 45.2%) was the most common type.

### Treatments and outcomes

The treatments and outcomes in both groups are summarized in Table 2. Medical treatments including blood pressure control, bowel rest, anticoagulation therapy, and antiplatelet therapy were indicated in both groups. Of the patients in the conservative group, 93.3% (14/15) experienced symptom relief. Only one patient had no improvement in symptoms after 7 days of treatment, and he later received endovascular treatment.

**Table 2:** Summary of treatments and outcomes in symptomatic SISMAD patients

	Conservative group (*n* = 15)	Endovascular group (*n* = 27)	*p* value
Median hospital stay (IQR), days	9 (3, 15)	9 (7, 11)	0.813
Blood pressure control, n (%)	10 (66.7)	13 (48.1)	0.337
Bowel rest, n (%)	8 (53.3)	18 (66.7)	0.511
Median fasting time (IQR), days	1 (0, 6)	1 (0, 5)	0.818
Anticoagulation therapy, n (%)	9 (60.0)	20 (74.1)	0.488
Antiplatelet therapy, n (%)	9 (60.0)	23 (85.2)	0.128
Technical success, n (%)	NA	23 (85.2)	/
Stent placement, n (%)	NA	23 (85.2)	/
Balloon angioplasty, n (%)	NA	7 (25.9)	/
Outcomes			
Symptom resolution, n (%)	14 (93.3)	26 (96.3)	1.000
^a^Complications, n (%)	0	1 (3.7)	1.000
Mortality, n (%)	0	0	1.000
^b^Follow-up			
Median time (IQR), months	25 (8, 55)	29 (17, 48)	0.333
Symptomatic, n (%)	6 (42.9)	1 (4.8)	< 0.001
Intestinal necrosis, n (%)	0	0	1.000
Underwent endovascular intervention, n (%)	4 (28.6)	NA	/
Secondary endovascular intervention, n (%)	NA	1 (4.8)	/
Disease unrelated mortality, n (%)	0	1 (4.8)	1.000
^c^Complete remodeling in the SMA, n (%)	1 (9.1)	^d^13 (86.7)	< 0.001

*IQR* interquartile range, *NA* Not applicable

^a^Includes one case of pseudoaneurysm formation in the left brachial artery

^b^The results of 14 patients in the conservative group and 21 patients in the endovascular group (Patients who had a failed endovascular intervention were excluded)

^c^The results of 11 patients in the conservative group and 15 patients in the endovascular group who had follow-up computed tomography angiography

^d^One patients had in-stent restenosis and one patient had occlusion in the distal SMA

The technical success rate in the endovascular group was 85.7% (23/27). The procedure failed in four patients because the guide wires could not enter the completely thrombosed true lumens (the details are summarized in Table 3). These four patients had medical treatment later, as they were not suspected of having bowel ischemia. The 23 patients with successful intervention had 37 bare metal stents deployed in total, 19 of whom had a single stent and 8 had overlapping stents. Seven patients had balloon angioplasty. Only one patient had abdominal pain 2 days after the intervention. However, the pain continued, and the cause was unclear after all examinations. There was only one case of a procedure-related complication: one patient had pseudoaneurysm formation in the left brachial artery. The patient had a second surgery for repair.

**Table 3:** Summary of the four patients who had a failed endovascular intervention

No.	Age-ranges	Sex	Comorbidity	Indication	Yun’s classification	Reason for failure	Treatment	Follow-up		
								Duration, months	Symptoms	Radiography
1	60–64	Male	Hypertension	Symptom recurrence after conservative treatment	IIb	Failure in true lumen cannulation	Endovascular→Conservative	12	None	Absent
2	60–64	Male	Diabetes mellitus	Persistent pain after conservative treatment	III	Failure in true lumen cannulation	Endovascular→Conservative	16	Intermittent abdominal pain	Newly appeared aneurysm
3	45–49	Male	None	Persistent pain after conservative treatment	III	Failure in true lumen cannulation	Endovascular→Conservative	16	Intermittent abdominal pain	Enriched collateral branches
4	45–49	Male	Hypertension	Persistent pain after conservative treatment	III	Failure in true lumen cannulation	Endovascular→Conservative	37	None	Increasing diameter in SMA

### Follow-up

There was a loss of contact with four patients in the follow-up period. Of the 38 patients with successful follow-up, the median time was 28.5 (15.5–48.0) months (Table 2). Six (42.9%) of the patients in the conservative group had recurrent symptoms. Symptoms included abdominal pain, diarrhea, and abdominal discomfort. Four of these symptomatic patients had endovascular intervention within less than 2 months after discharge, and none of them had any symptom recurrence after the interventions.

**Fig. 1:**
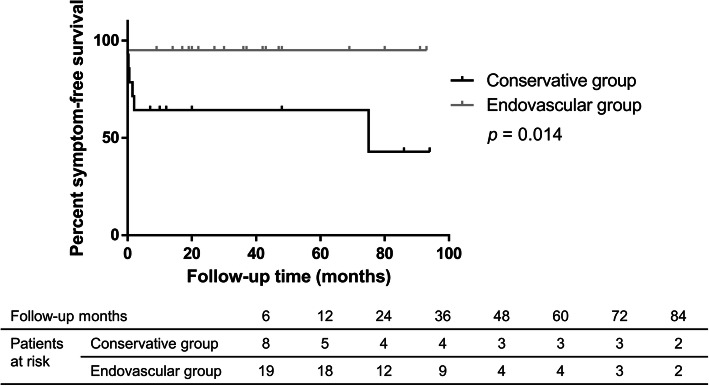
Kaplan-Meier curve for symptom-free survival in SISMAD

Among the patients who had a successful endovascular procedure, only 1 patient had recurrent symptoms during follow-up. He had a second endovascular intervention because severe in-stent restenosis was observed. One patient in the endovascular group died due to an unrelated reason. The symptom-free survival rates of both groups are shown in Fig. 1. The endovascular group had a significantly longer time of symptom-free survival (*p* = 0.014). The 1-year event-free survival percentages were 64.3% in the conservative group and 95.0% in the endovascular group.

**Fig. 2:**
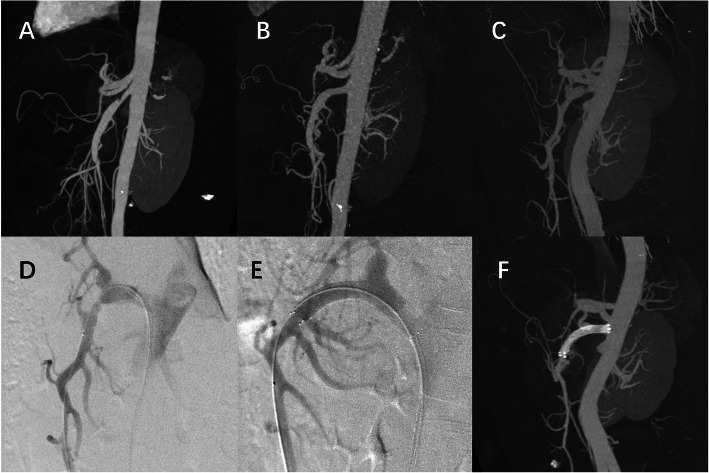
Two cases of SISMAD patients. **a** & **b** A patient of Yun’s type IIa who had incomplete remodeling after 1 year of conservative treatment (**a** before treatment; **b** 1 year after discharge). **c**, **d**, **e**, and **f** A patient of Yun’s type IIa who had successful endovascular treatment and showed good stent patency during follow-up (**c** & **d** CTA and digital subtraction angiography [DSA] before the intervention; **e** DSA after stent implantation; **f** follow-up CTA after 2 years)

Eleven patients in the conservative group and 18 patients in the endovascular group had radiographic examinations during the follow-up period. Figure 2 shows two cases from the different groups. In the conservative group, complete remodeling appeared in only one patient. However, among the patients with successful stent placement, 86.7% (13/15) had complete remodeling in the SMA, which was significantly higher than the conservative group (*p* < 0.001).

Of the four patients who failed in the endovascular treatment, none had additional invasive treatment during follow-up. Two of them had symptom recurrence, and two had morphological progression (Table 3). Because there was no evidence of bowel ischemia, medical treatment was continued under close surveillance.

## Discussion

Due to the low prevalence of SISMAD, the treatment strategy is still under debate. Some patients have severe complications, such as intestinal necrosis; in such cases, open surgery is inevitable. Conservative treatment is recommended as the first-line therapy for most patients [3]. However, some patients had symptom recurrence after conservative treatment, and some required additional intervention [5]. Endovascular treatment has shown favorable outcomes, in both the perioperative and follow-up periods [2, 12]. The indications of endovascular treatment should be further investigated.

With the development of imaging techniques, SISMAD is increasingly being recognized, but the aetiology is unknown. The disease seems to be associated with male gender (90%), hypertension (41%), and smoking (45%) [5]. However, the prevalence of diabetes mellitus was relatively low (8%) [3]. Min and his colleagues compared the occurrence sites of the dissections and atherosclerotic plaques of SMA. The vast differences showed the opposite aetiology of atherosclerotic plaques and dissections [13]. The disease might also be associated with the morphology of SMA, and a case-control study demonstrated that SISMAD patients had larger mean SMA-distal aorta angles than healthy volunteers [14]. We observed that 60% of our patients had hyperhomocysteinemia, which might be another risk factor for SISMAD. In addition, SISMAD patients require some radiographic examinations to confirm if their conditions are combined with other vascular diseases.

Radiographic results are associated with the severity of SISMAD to some extent. Symptomatic patients had longer lengths of dissections and shorter diameters of true lumens [15]. Currently, Sakamoto’s and Yun’s classifications are proposed to assess SISMAD [11, 16]. Yun’s classification [11] mainly focus on the patency of SMA, and both true lumen and false lumen are included. Studies have shown that Yun’s type IIb comprises approximately half of SISMAD patients [5] and is the most common type. In addition, stenosis or occlusion in the true lumen, dissection aneurysm, and branch involvement were frequently observed in our patients. These radiographic features could provide guidance on the treatment strategy, which should be further studied.

Initial conservative treatment is safe for asymptomatic SISMAD patients [5]. In symptomatic patients, although conservative treatment is an effective way to relieve symptoms [9, 15, 17, 18], the outcomes are not satisfactory in the long term. From 12.3 to 18.1% of the initial conservatively treated patients required additional treatments (endovascular treatment or open surgery) [19–21]. Follow-up radiography also suggests that conservative treatment alone may not be able to cure the disease. As Wang reported, only 25% of the SMAD patients had complete remodeling during follow-up, and 12% showed morphologic progression [5]. In our experience, 42.9% of the patients in the conservative group had symptom recurrence during follow-up, and 28.6% had additional endovascular treatment. Therefore, close surveillance was necessary for these patients.

Due to the high rate of symptom recurrence in the conservative group, more evidence is needed to identify the high-risk patients. According to our experience, severe true lumen stenosis or occlusion might be an important risk factor. A total of 66.6%(6/9) of the patients in the conservative group who had severe true lumen stenosis or occlusion had symptom recurrence during follow-up; however, none of the other 5 patients had symptom recurrence (*p* = 0.031). Li and Qiu proposed their modified classifications, where dissections with severe true lumen stenosis or occlusion were classified into different subtypes [2, 22]. Severe true lumen stenosis was considered as an important indication of endovascular intervention in some studies [6, 23, 24]. However, there are no robust data on the associations between the radiographic results and prognosis, which remain to be further investigated.

The indications of endovascular treatment include persistent pain, aneurysm progression, and suspicion of bowel ischemia [5, 18, 25, 26]. In our center, endovascular treatment was also indicated in patients who had symptom recurrence after conservative treatment. Endovascular treatment was both safe and effective. Procedure-related complications have rarely been reported in the literature [10, 12, 27]. After the intervention, symptoms disappeared in most patients, even in those with extremely severe symptoms.

Endovascular intervention in SISMAD patients had a relatively high rate of technical success [22, 28, 29]. There were four cases of technical failure, and all of them were associated with the failure of true lumen cannulation. In accordance with the previous studies [27], severe true lumen stenosis or complete occlusion might be the most common reason for technical failure. In the long term, these patients would be at high risk of symptom recurrence, and open bypass surgery might be the next step [3].

The long-term outcomes of endovascular treatment have been reported in several studies in the literature [7, 27, 30]. Long-term complications included stent stenosis, stent occlusion, and stent thrombosis [7, 8, 27]. Endothelial injury, neointimal hyperplasia, and chronic inflammation could be the causes [31, 32]. However, the incidence was relatively low, and only 2.1% of the endovascularly treated patients had a secondary intervention [5]. In our experience, only one patient had in-stent restenosis and had stent implantation for the second time. None of the remaining 20 patients with successful stenting had recurrent symptoms. These results showed the good prognosis of endovascularly treated patients.

The symptom-free survival of the two groups was significantly different (Fig. 1, *p* = 0.014). Although both treatments had a high rate of symptom relief, conservatively treated patients were more likely to have recurrent symptoms during follow-up. Furthermore, patients in the endovascular group had a higher rate of SMA remodeling (*p* < 0.001). These results indicate that endovascularly treated patients had better outcomes in the long term, suggesting that endovascular treatment might also be an appropriate first-line treatment for symptomatic SISMAD patients.

The limitations of this study are as follows. 1) It was a retrospective study, and all data were collected from a single center. 2) Some patients did not have any imaging examinations during follow-up. 3) The potential associations between the radiographic findings and prognosis were not analysed. Further studies are needed.

## Conclusions

To conclude, both conservative treatment and endovascular treatment can effectively relieve the symptoms of SISMAD patients in the short-term. In the long term, compared with conservative treatment, endovascular treatment had a lower rate of symptom recurrence and a higher rate of complete remodeling. Prospective, multi-center studies are needed to confirm the long-term outcomes of both treatments.

## Data Availability

The datasets generated and analyzed during the current study are available from the corresponding author on reasonable request.

## Abbreviations

SISMAD: Spontaneous isolated superior mesenteric artery dissection;
ESVS: European Society of Vascular Surgery;
CTA: Computed tomography angiography;
SMA: Superior mesenteric artery.

## References

[1] Bauersfeld SR (1947). Dissecting aneurysm of the aorta; a presentation of 15 cases and a review of the recent literature. Ann Intern Med.

[2] Qiu C, He Y, Li D, Shang T, Wang X, Wu Z (2019). Mid-term results of endovascular treatment for spontaneous isolated dissection of the superior mesenteric artery. Eur J Vasc Endovasc Surg.

[3] Bjorck M, Koelemay M, Acosta S, Bastos Goncalves F, Kolbel T, Kolkman JJ (2017). Editor's choice - Management of the Diseases of mesenteric arteries and veins: clinical practice guidelines of the European Society of Vascular Surgery (ESVS). Eur J Vasc Endovasc Surg.

[4] Garrett HE (2014). Options for treatment of spontaneous mesenteric artery dissection. J Vasc Surg.

[5] Wang J, He Y, Zhao J, Yuan D, Xu H, Ma Y (2018). Systematic review and meta-analysis of current evidence in spontaneous isolated celiac and superior mesenteric artery dissection. J Vasc Surg.

[6] Jia ZZ, Zhao JW, Tian F, Li SQ, Wang K, Wang Y (2013). Initial and middle-term results of treatment for symptomatic spontaneous isolated dissection of superior mesenteric artery. Eur J Vasc Endovasc Surg.

[7] Kim J, Yoon CJ, Seong N, Lee H, Kim YJ (2017). Spontaneous dissection of superior mesenteric artery: long-term outcome of stent placement. J Vasc Interv Radiol.

[8] Wen D, Wang Z, Yu J, Zhang W, Yang X, He H (2018). Endovascular stent-graft repair of spontaneous isolated dissection of the superior mesenteric artery. Cardiovasc Intervent Radiol.

[9] Tomita K, Obara H, Sekimoto Y, Matsubara K, Watada S, Fujimura N (2016). Evolution of computed tomographic characteristics of spontaneous isolated superior mesenteric artery dissection during conservative management. Circ J.

[10] Pang P, Jiang Z, Huang M, Zhou B, Zhu K, Shan H (2013). Value of endovascular stent placement for symptomatic spontaneous isolated superior mesenteric artery dissection. Eur J Radiol.

[11] Yun WS, Kim YW, Park KB, Cho SK, Do YS, Lee KB (2009). Clinical and angiographic follow-up of spontaneous isolated superior mesenteric artery dissection. Eur J Vasc Endovasc Surg.

[12] Jia Z, Su H, Chen W, Ni G, Qi C, Gu J (2019). Endovascular treatment of patients with isolated mesenteric artery dissection aneurysm: bare stents alone versus stent assisted coiling. Eur J Vasc Endovasc Surg.

[13] Min ZG, Shan HR, Xu L, Yan S, Sheng XX, Ji J (2017). Spontaneous isolated dissection and atherosclerotic plaques of superior mesenteric artery: the vastly different occurrence site suggests the opposite haemodynamic aetiology. Br J Radiol.

[14] Wu Z, Yi J, Xu H, Guo W, Wang L, Chen D (2017). The significance of the angle between superior mesenteric artery and aorta in spontaneous isolated superior mesenteric artery dissection. Ann Vasc Surg.

[15] Zhang X, Xiang P, Yang Y, Chen J, Guan J, Liu M (2018). Correlation between computed tomography features and clinical presentation and Management of Isolated Superior Mesenteric Artery Dissection. Eur J Vasc Endovasc Surg.

[16] Sakamoto I, Ogawa Y, Sueyoshi E, Fukui K, Murakami T, Uetani M (2007). Imaging appearances and management of isolated spontaneous dissection of the superior mesenteric artery. Eur J Radiol.

[17] Heo SH, Kim YW, Woo SY, Park YJ, Park KB, Kim DK (2017). Treatment strategy based on the natural course for patients with spontaneous isolated superior mesenteric artery dissection. J Vasc Surg.

[18] Kim H, Park H, Park SJ, Park BW, Hwang JC, Seo YW (2018). Outcomes of spontaneous isolated superior mesenteric artery dissection without antithrombotic use. Eur J Vasc Endovasc Surg.

[19] Kimura Y, Kato T, Inoko M (2018). Outcomes of treatment strategies for isolated spontaneous dissection of the superior mesenteric artery: a systematic review. Ann Vasc Surg.

[20] Zhu Y, Peng Y, Xu M, Wei Y, Wu S, Guo W (2018). Treatment strategies and outcomes of symptomatic spontaneous isolated superior mesenteric artery dissection: a systematic review and meta-analysis. J Endovasc Ther.

[21] Karaolanis G, Antonopoulos C, Tsilimigras DI, Moris D, Moulakakis K (2019). Spontaneous isolated superior mesenteric artery dissection. Systematic review and meta-analysis. Vascular.

[22] Li DL, He YY, Alkalei AM, Chen XD, Jin W, Li M (2014). Management strategy for spontaneous isolated dissection of the superior mesenteric artery based on morphologic classification. J Vasc Surg.

[23] Cho BS, Lee MS, Lee MK, Choi YJ, Kim CN, Kang YJ (2011). Treatment guidelines for isolated dissection of the superior mesenteric artery based on follow-up CT findings. Eur J Vasc Endovasc Surg.

[24] Min SI, Yoon KC, Min SK, Ahn SH, Jae HJ, Chung JW (2011). Current strategy for the treatment of symptomatic spontaneous isolated dissection of superior mesenteric artery. J Vasc Surg.

[25] Loeffler JW, Obara H, Fujimura N, Bove P, Newton DH, Zettervall SL (2017). Medical therapy and intervention do not improve uncomplicated isolated mesenteric artery dissection outcomes over observation alone. J Vasc Surg.

[26] Kimura Y, Kato T, Nagao K, Izumi T, Haruna T, Ueyama K (2017). Outcomes and radiographic findings of isolated spontaneous superior mesenteric artery dissection. Eur J Vasc Endovasc Surg.

[27] Dong Z, Ning J, Fu W, Guo D, Xu X, Chen B (2016). Failures and lessons in the endovascular treatment of symptomatic isolated dissection of the superior mesenteric artery. Ann Vasc Surg.

[28] Luan JY, Li X (2013). Computed tomography imaging features and classification of isolated dissection of the superior mesenteric artery. Eur J Vasc Endovasc Surg.

[29] Li N, Lu QS, Zhou J, Bao JM, Zhao ZQ, Jing ZP (2014). Endovascular stent placement for treatment of spontaneous isolated dissection of the superior mesenteric artery. Ann Vasc Surg.

[30] Dong Z, Fu W, Chen B, Guo D, Xu X, Wang Y (2013). Treatment of symptomatic isolated dissection of superior mesenteric artery. J Vasc Surg.

[31] Scott RA, Panitch A (2014). Macromolecular approaches to prevent thrombosis and intimal hyperplasia following percutaneous coronary intervention. Biomacromolecules.

[32] Brancati MF, Burzotta F, Trani C, Leonzi O, Cuccia C, Crea F (2017). Coronary stents and vascular response to implantation: literature review. Pragmat Obs Res.

